# *N*′-[(*E*)-1-(4-Chloro­phen­yl)ethyl­idene]-2-[4-(2-methyl­prop­yl)phen­yl]propano­hydrazide

**DOI:** 10.1107/S1600536808039226

**Published:** 2008-12-03

**Authors:** Hoong-Kun Fun, Samuel Robinson Jebas, K. V Sujith, B. Kalluraya

**Affiliations:** a X-ray Crystallography Unit, School of Physics, Universiti Sains Malaysia, 11800 USM, Penang, Malaysia; b Department of Studies in Chemistry, Mangalore University, Mangalagangotri, Mangalore 574 199, India

## Abstract

The asymmetric unit of the title compound, C_21_H_25_ClN_2_O, contains four crystallographically independent mol­ecules, which differ mainly in the orientation of the isobutyl groups. The benzene rings are almost orthogonal to each other, forming dihedral angles of 87.40 (6), 88.69 (6), 84.88 (6) and 85.12 (6)° in the four mol­ecules. The crystal structure is stabilized by inter­molecular N—H⋯O and C—H⋯O hydrogen bonds, together with C—H⋯π inter­actions.

## Related literature

For the synthesis of pyrazolines and pyrazoles, see: Sridhar & Perumal (2003 ▶). For the pharmaceutical applications of hydrazones, see: Bedia *et al.* (2006 ▶); Rollas *et al.* (2002 ▶); Terzioglu & Gursoy (2003 ▶). For related literature on ibuprofen, see: Amir & Kumar (2007 ▶). For bond-length data, see: Allen *et al.* (1987 ▶).
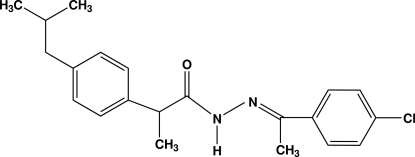


## Experimental

### 

#### Crystal data

C_21_H_25_ClN_2_O
*M* *_r_* = 356.88
Triclinic, 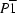

*a* = 14.2691 (2) Å
*b* = 15.5343 (2) Å
*c* = 20.7436 (3) Å
α = 77.416 (1)°
β = 90.058 (1)°
γ = 62.714 (1)°
*V* = 3961.17 (10) Å^3^
*Z* = 8
Mo *K*α radiation
μ = 0.20 mm^−1^
*T* = 100.0 (1) K
0.54 × 0.18 × 0.17 mm

#### Data collection

Bruker SMART APEXII CCD area-detector diffractometer
Absorption correction: multi-scan (*SADABS*; Bruker, 2005 ▶) *T*_min_ = 0.899, *T*_max_ = 0.966
132549 measured reflections
28009 independent reflections
19617 reflections with *I* > 2σ(*I*)
*R*_int_ = 0.040

#### Refinement

*R*[*F* ^2^ > 2σ(*F* ^2^)] = 0.065
*wR*(*F* ^2^) = 0.166
*S* = 1.08
28009 reflections
933 parameters
4 restraints
H atoms treated by a mixture of independent and constrained refinement
Δρ_max_ = 0.65 e Å^−3^
Δρ_min_ = −0.28 e Å^−3^

### 

Data collection: *APEX2* (Bruker, 2005 ▶); cell refinement: *SAINT* (Bruker, 2005 ▶); data reduction: *SAINT*; program(s) used to solve structure: *SHELXTL* (Sheldrick, 2008 ▶); program(s) used to refine structure: *SHELXTL*; molecular graphics: *SHELXTL*; software used to prepare material for publication: *SHELXTL* and *PLATON* (Spek, 2003 ▶).

## Supplementary Material

Crystal structure: contains datablocks global, I. DOI: 10.1107/S1600536808039226/rz2270sup1.cif

Structure factors: contains datablocks I. DOI: 10.1107/S1600536808039226/rz2270Isup2.hkl

Additional supplementary materials:  crystallographic information; 3D view; checkCIF report

## Figures and Tables

**Table 1:** Hydrogen-bond geometry (Å, °) *Cg*1, *Cg*2, *Cg*3 and *Cg*4 are the centroids of the C10*A*–C15*A*, C10*B*–C15*B*, C10*C*–C15*C* and C10*D*–C15*D* rings, respectively.

*D*—H⋯*A*	*D*—H	H⋯*A*	*D*⋯*A*	*D*—H⋯*A*
N2*A*—H2*NA*⋯O1*B*^i^	0.847 (14)	2.090 (17)	2.9255 (19)	169 (3)
N2*B*—H2*NB*⋯O1*A*^i^	0.852 (18)	2.069 (18)	2.916 (2)	173 (2)
N2*C*—H2*NC*⋯O1*D*^ii^	0.845 (15)	2.075 (17)	2.9085 (19)	169 (3)
N2*D*—H2*ND*⋯O1*C*^iii^	0.855 (16)	2.085 (17)	2.934 (2)	172 (3)
C20*B*—H20*A*⋯O1*A*^i^	0.96	2.47	3.125 (3)	126
C20*C*—H20*D*⋯O1*D*^ii^	0.96	2.57	3.099 (3)	115
C20*D*—H20*G*⋯O1*C*^ii^	0.96	2.50	3.175 (3)	128
C2*OA*—H2*OC*⋯*Cg*1^iv^	0.96	2.75	3.4628 (19)	131
C16*C*—H16*F*⋯*Cg*2	0.96	2.78	3.744 (2)	171
C20*B*—H20*C*⋯*Cg*2^i^	0.96	2.94	3.5787 (19)	125
C16*A*—H16*B*⋯*Cg*3^v^	0.96	2.90	3.860 (2)	170
C20*C*—H20*F*⋯*Cg*3^ii^	0.96	2.77	3.4943 (19)	132
C16*B*—H16*D*⋯*Cg*4	0.96	2.86	3.818 (2)	171
C20*D*—H20*I*⋯*Cg*4^vi^	0.96	2.96	3.5557 (19)	122

Symmetry codes: (i) 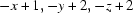; (ii) 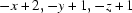; (iii) 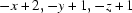; (iv) 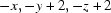; (v) 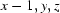; (vi) 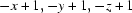.

**Fig. 1.**
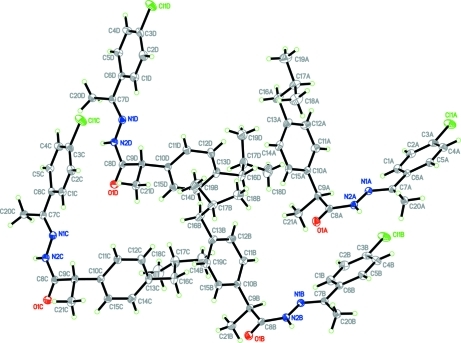
The molecular structure of the title compound, showing 50% probability displacement ellipsoids and the atom numbering scheme.

**Fig. 2.**
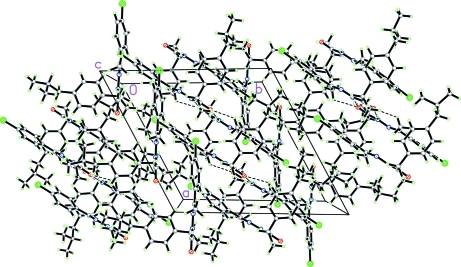
The crystal packing of the title compound, viewed down the c axis. Intermolecular hydrogen bonds are shown as dashed lines.

## supplementary crystallographic information

### Comment

Aryl hydrazones are important building blocks for the synthesis of a variety of heterocyclic compounds such as pyrazolines and pyrazoles (Sridhar & Perumal, 2003). Aryl hydrazones have been most conveniently synthesized by the reaction of aryl hydrazines with carbonyl compounds. The reaction has been performed in a variety of solvents such as ethanol, acetic acid, dioxan, diglyme, DMF and others with or without an acidic catalyst. Hydrazones possessing an azometine –NHN═CH– group constitute an important class of compounds for new drug development. Therefore many researchers have synthesized these compounds as target structures and evaluated their biological activities. Hydrazones have been demonstrated to possess antimicrobial, anticonvulsant, analgesic, anti-inflammatory, antiplatelet, antitubercular, anticancer and antitumoral activities (Bedia *et al.*, 2006; Rollas *et al.*, 2002; Terzioglu & Gursoy, 2003). These observations have been the guide for the development of new hydrazones that possess various biological activities. Similarly ibuprofen is also known for its pharmaceutical activities and belongs to the class of non-steroidal anti-inflammatory Drugs (Amir & Kumar, 2007). Prompted by these review, the title compound was synthesized and its crystal structure is reported here.

The asymmetric unit of the title compound (Fig. 1) contains four crystallographically independent molecules (*A*, *B*, *C* & *D*) with similar geometries, the main difference consisting in the orientation of the isobutyl groups. Bond lengths (Allen *et al.*, 1987) and angles have normal values. The benzene rings in each molecule are almost orthogonal to each other, as indicated by the dihedral angles of 87.40 (6)° (C1A—C6A; C10A—C15A), 88.69 (6)° (C1B—C6B; C10B—C15B), 84.88 (6)° (C1C—C6C; C10C—C15C) and 85.12 (6)° (C1D—C6D; C10D—C15D). The crystal structure (Fig. 2) is stabilized by intermolecular N—H···O and C—H···O hydrogen bonds together with C—H···π interactions (Table 1).

It is worth noting that the unit cell adopted in the present structure analysis is a superstructure of a fundamental monoclinic unit cell (cell 2) with half of the volume of the experimentally determined triclinic cell (cell 1). The two cells are related by the following transformation matrix: [*a*, *b*, *c*]_cell 2_ = [1/2, 1, 0, 1/2, 0 0, 0,0, 1] [*a*, *b*, *c*]_cell 1_. The dimensions of the fundamental cell are: *a* = 13.8052 (2), *b* = 7.1345 (2), *c* = 20.7436 (3) Å, *β* = 104.224 (1)°; space group *P*2_1_/*n*. Solution and refinement of the structure in cell 2 were not successful.

### Experimental

The title compound was obtained by refluxing 2-[4-(2-methylpropyl)phenyl]propanehydrazide (0.01 mol) and 4-chloroacetophenone (0.01 mol) in ethanol (30 ml) by adding 3 drops of concentrated sulfuric acid for 1 h. Excess ethanol was removed from the reaction mixture under reduced pressure. The solid product obtained was filtered, washed with ethanol and dried. Crystals suitable for X-ray analysis were obtained by slow evaporation of an ethanol solution (yield 87%; m.p.418 K, MW 356).

### Refinement

N-bound H atoms were located in a difference map and were refined with the N—H bond lengths restrained to 0.85 (1) Å. Other H atoms were positioned geometrically and refined using a riding model, with C—H=0.93–0.98 Å and with *U*_iso_(H) = 1.2 U_eq_(C) or 1.5 U_eq_(C) for methyl H atoms. A rotating-group model was used for the methyl groups.

### Crystal data

**Table:**

C_21_H_25_ClN_2_O	*Z* = 8
*M**_r_* = 356.88	*F*(000) = 1520
Triclinic, *P*1	*D*_x_ = 1.197 Mg m^−^^3^
Hall symbol: -P 1	Mo *K*α radiation, λ = 0.71073 Å
*a* = 14.2691 (2) Å	Cell parameters from 9864 reflections
*b* = 15.5343 (2) Å	θ = 2.7–32.1°
*c* = 20.7436 (3) Å	µ = 0.20 mm^−^^1^
α = 77.416 (1)°	*T* = 100 K
β = 90.058 (1)°	Block, colourless
γ = 62.714 (1)°	0.54 × 0.18 × 0.17 mm
*V* = 3961.17 (10) Å^3^	

### Data collection

**Table:**

Bruker SMART APEXII CCD area-detector diffractometer	28009 independent reflections
Radiation source: fine-focus sealed tube	19617 reflections with *I* > 2σ(*I*)
graphite	*R*_int_ = 0.040
φ and ω scans	θ_max_ = 32.3°, θ_min_ = 1.0°
Absorption correction: multi-scan (*SADABS*; Bruker, 2005)	*h* = −20→21
*T*_min_ = 0.899, *T*_max_ = 0.966	*k* = −23→23
132549 measured reflections	*l* = −31→31

### Refinement

**Table:**

Refinement on *F*^2^	Primary atom site location: structure-invariant direct methods
Least-squares matrix: full	Secondary atom site location: difference Fourier map
*R*[*F*^2^ > 2σ(*F*^2^)] = 0.065	Hydrogen site location: inferred from neighbouring sites
*wR*(*F*^2^) = 0.166	H atoms treated by a mixture of independent and constrained refinement
*S* = 1.08	*w* = 1/[σ^2^(*F*_o_^2^) + (0.0489*P*)^2^ + 3.7993*P*] where *P* = (*F*_o_^2^ + 2*F*_c_^2^)/3
28009 reflections	(Δ/σ)_max_ = 0.001
933 parameters	Δρ_max_ = 0.65 e Å^−^^3^
4 restraints	Δρ_min_ = −0.28 e Å^−^^3^

### Special details

**Table:**

Experimental. The data was collected with the Oxford Cyrosystem Cobra low-temperature attachment.
Geometry. All e.s.d.'s (except the e.s.d. in the dihedral angle between two l.s. planes) are estimated using the full covariance matrix. The cell e.s.d.'s are taken into account individually in the estimation of e.s.d.'s in distances, angles and torsion angles; correlations between e.s.d.'s in cell parameters are only used when they are defined by crystal symmetry. An approximate (isotropic) treatment of cell e.s.d.'s is used for estimating e.s.d.'s involving l.s. planes.
Refinement. Refinement of *F*^2^ against ALL reflections. The weighted *R*-factor *wR* and goodness of fit *S* are based on *F*^2^, conventional *R*-factors *R* are based on *F*, with *F* set to zero for negative *F*^2^. The threshold expression of *F*^2^ > σ(*F*^2^) is used only for calculating *R*-factors(gt) *etc*. and is not relevant to the choice of reflections for refinement. *R*-factors based on *F*^2^ are statistically about twice as large as those based on *F*, and *R*- factors based on ALL data will be even larger.

### Fractional atomic coordinates and isotropic or equivalent isotropic displacement parameters (Å^2^)

**Table:**

	*x*	*y*	*z*	*U*_iso_*/*U*_eq_	
Cl1A	−0.50215 (4)	1.34130 (4)	0.82547 (3)	0.03036 (11)	
O1A	0.29382 (10)	0.96358 (10)	0.92933 (6)	0.0218 (2)	
N1A	0.01803 (11)	1.09790 (10)	0.91770 (7)	0.0160 (2)	
N2A	0.12260 (11)	1.04811 (10)	0.94423 (7)	0.0158 (2)	
C1A	−0.18634 (14)	1.22066 (13)	0.85444 (8)	0.0194 (3)	
H1AA	−0.1309	1.2090	0.8283	0.023*	
C2A	−0.28962 (14)	1.26671 (13)	0.82420 (9)	0.0209 (3)	
H2AA	−0.3037	1.2860	0.7781	0.025*	
C3A	−0.37220 (14)	1.28381 (13)	0.86357 (9)	0.0201 (3)	
C4A	−0.35268 (14)	1.25639 (13)	0.93217 (9)	0.0201 (3)	
H4AA	−0.4085	1.2685	0.9580	0.024*	
C5A	−0.24866 (13)	1.21050 (12)	0.96211 (8)	0.0176 (3)	
H5AA	−0.2352	1.1923	1.0083	0.021*	
C6A	−0.16400 (13)	1.19132 (11)	0.92377 (8)	0.0151 (3)	
C7A	−0.05227 (13)	1.13919 (11)	0.95537 (8)	0.0153 (3)	
C8A	0.20007 (13)	1.00697 (12)	0.90573 (8)	0.0160 (3)	
C9A	0.16750 (13)	1.01376 (12)	0.83402 (8)	0.0161 (3)	
H9AA	0.0980	1.0726	0.8187	0.019*	
C10A	0.15797 (13)	0.92062 (12)	0.83157 (7)	0.0159 (3)	
C11A	0.06320 (14)	0.92817 (13)	0.80610 (8)	0.0205 (3)	
H11A	0.0051	0.9909	0.7901	0.025*	
C12A	0.05463 (16)	0.84261 (14)	0.80440 (9)	0.0238 (3)	
H12A	−0.0090	0.8494	0.7867	0.029*	
C13A	0.13929 (16)	0.74756 (14)	0.82873 (8)	0.0227 (3)	
C14A	0.23477 (15)	0.74057 (13)	0.85333 (9)	0.0221 (3)	
H14A	0.2931	0.6779	0.8690	0.027*	
C15A	0.24412 (14)	0.82537 (12)	0.85485 (8)	0.0185 (3)	
H15A	0.3083	0.8186	0.8715	0.022*	
C16A	0.12727 (18)	0.65540 (15)	0.82993 (10)	0.0288 (4)	
H16A	0.1967	0.6004	0.8291	0.035*	
H16B	0.0832	0.6677	0.7899	0.035*	
C17A	0.07800 (17)	0.62367 (15)	0.89080 (10)	0.0279 (4)	
H17A	0.0100	0.6808	0.8930	0.033*	
C18A	0.1489 (2)	0.59176 (18)	0.95570 (11)	0.0389 (5)	
H18A	0.1148	0.5740	0.9923	0.058*	
H18B	0.1607	0.6460	0.9610	0.058*	
H18C	0.2156	0.5352	0.9546	0.058*	
C19A	0.0569 (2)	0.53832 (17)	0.88258 (12)	0.0392 (5)	
H19A	0.0113	0.5592	0.8421	0.059*	
H19B	0.0233	0.5209	0.9196	0.059*	
H19C	0.1230	0.4813	0.8809	0.059*	
C2OA	−0.02665 (13)	1.13534 (13)	1.02674 (8)	0.0182 (3)	
H2OA	0.0243	1.1591	1.0293	0.027*	
H2OB	0.0024	1.0677	1.0529	0.027*	
H2OC	−0.0903	1.1767	1.0435	0.027*	
C21A	0.24880 (15)	1.02591 (14)	0.78999 (9)	0.0229 (3)	
H21A	0.2546	1.0836	0.7944	0.034*	
H21B	0.2261	1.0343	0.7444	0.034*	
H21C	0.3166	0.9675	0.8035	0.034*	
Cl1B	−0.01617 (4)	1.34985 (4)	0.82882 (3)	0.03232 (11)	
O1B	0.78289 (10)	0.97763 (10)	0.92321 (6)	0.0212 (2)	
N1B	0.50620 (11)	1.10321 (10)	0.91489 (7)	0.0165 (3)	
N2B	0.61177 (11)	1.05501 (11)	0.94034 (7)	0.0167 (3)	
C1B	0.29988 (15)	1.22571 (14)	0.85348 (8)	0.0218 (3)	
H1BA	0.3543	1.2136	0.8265	0.026*	
C2B	0.19567 (15)	1.27267 (14)	0.82468 (9)	0.0244 (4)	
H2BA	0.1801	1.2917	0.7786	0.029*	
C3B	0.11476 (14)	1.29114 (13)	0.86498 (9)	0.0214 (3)	
C4B	0.13641 (14)	1.26365 (14)	0.93363 (9)	0.0224 (3)	
H4BA	0.0815	1.2767	0.9603	0.027*	
C5B	0.24147 (14)	1.21619 (13)	0.96217 (8)	0.0202 (3)	
H5BA	0.2564	1.1976	1.0082	0.024*	
C6B	0.32483 (13)	1.19603 (12)	0.92300 (8)	0.0160 (3)	
C7B	0.43685 (13)	1.14461 (12)	0.95318 (8)	0.0157 (3)	
C8B	0.68886 (13)	1.01664 (12)	0.90082 (8)	0.0158 (3)	
C9B	0.65608 (13)	1.01972 (12)	0.83002 (8)	0.0162 (3)	
H9BA	0.5834	1.0738	0.8158	0.019*	
C10B	0.65861 (13)	0.92095 (12)	0.82936 (7)	0.0154 (3)	
C11B	0.56975 (14)	0.91906 (12)	0.80348 (8)	0.0183 (3)	
H11B	0.5075	0.9786	0.7877	0.022*	
C12B	0.57317 (15)	0.82861 (13)	0.80093 (9)	0.0217 (3)	
H12B	0.5135	0.8288	0.7826	0.026*	
C13B	0.66450 (15)	0.73792 (13)	0.82544 (8)	0.0200 (3)	
C14B	0.75330 (14)	0.74044 (13)	0.85116 (9)	0.0208 (3)	
H14B	0.8152	0.6809	0.8676	0.025*	
C15B	0.75094 (14)	0.83051 (12)	0.85259 (8)	0.0185 (3)	
H15B	0.8115	0.8305	0.8692	0.022*	
C16B	0.66861 (17)	0.63894 (13)	0.82619 (9)	0.0245 (4)	
H16C	0.7388	0.5935	0.8175	0.029*	
H16D	0.6180	0.6488	0.7905	0.029*	
C17B	0.64403 (16)	0.59068 (14)	0.89191 (9)	0.0253 (4)	
H17B	0.6887	0.5895	0.9283	0.030*	
C18B	0.52867 (19)	0.65016 (18)	0.90218 (13)	0.0390 (5)	
H18D	0.5128	0.7179	0.9001	0.059*	
H18E	0.5164	0.6209	0.9449	0.059*	
H18F	0.4838	0.6494	0.8680	0.059*	
C19B	0.6697 (2)	0.48320 (15)	0.89375 (12)	0.0372 (5)	
H19D	0.7433	0.4456	0.8885	0.056*	
H19E	0.6265	0.4830	0.8583	0.056*	
H19F	0.6554	0.4534	0.9356	0.056*	
C20B	0.46283 (14)	1.14388 (14)	1.02360 (8)	0.0206 (3)	
H20A	0.5209	1.1587	1.0259	0.031*	
H20B	0.4823	1.0791	1.0520	0.031*	
H20C	0.4019	1.1933	1.0379	0.031*	
C21B	0.73054 (16)	1.04037 (14)	0.78289 (9)	0.0233 (3)	
H21D	0.7093	1.0426	0.7384	0.035*	
H21E	0.8021	0.9881	0.7967	0.035*	
H21F	0.7267	1.1033	0.7841	0.035*	
Cl1C	0.83776 (4)	0.15525 (4)	0.67181 (3)	0.03310 (11)	
O1C	1.25756 (10)	0.53449 (10)	0.57579 (6)	0.0220 (2)	
N1C	1.11575 (11)	0.39962 (10)	0.58593 (7)	0.0164 (3)	
N2C	1.17065 (11)	0.45005 (11)	0.56011 (7)	0.0168 (3)	
C1C	1.03591 (14)	0.27335 (13)	0.64667 (8)	0.0189 (3)	
H1CA	1.0807	0.2829	0.6736	0.023*	
C2C	0.97822 (14)	0.22643 (13)	0.67556 (9)	0.0213 (3)	
H2CA	0.9844	0.2046	0.7216	0.026*	
C3C	0.91144 (14)	0.21232 (13)	0.63540 (9)	0.0208 (3)	
C4C	0.90208 (14)	0.24352 (13)	0.56681 (9)	0.0212 (3)	
H4CA	0.8573	0.2334	0.5402	0.025*	
C5C	0.96042 (13)	0.29019 (12)	0.53803 (8)	0.0183 (3)	
H5CA	0.9548	0.3108	0.4920	0.022*	
C6C	1.02740 (13)	0.30639 (12)	0.57749 (8)	0.0158 (3)	
C7C	1.08685 (13)	0.35933 (12)	0.54719 (8)	0.0156 (3)	
C8C	1.20567 (13)	0.49209 (12)	0.59893 (8)	0.0163 (3)	
C9C	1.17627 (13)	0.48785 (12)	0.66953 (8)	0.0166 (3)	
H9CA	1.1647	0.4296	0.6848	0.020*	
C10C	1.07335 (13)	0.58187 (12)	0.66935 (7)	0.0154 (3)	
C11C	0.98370 (14)	0.57633 (13)	0.69243 (8)	0.0190 (3)	
H11C	0.9865	0.5142	0.7078	0.023*	
C12C	0.89064 (14)	0.66244 (13)	0.69266 (8)	0.0216 (3)	
H12C	0.8324	0.6569	0.7088	0.026*	
C13C	0.88253 (14)	0.75716 (13)	0.66922 (8)	0.0209 (3)	
C14C	0.97276 (15)	0.76233 (13)	0.64644 (9)	0.0213 (3)	
H14C	0.9699	0.8245	0.6310	0.026*	
C15C	1.06656 (14)	0.67620 (13)	0.64655 (8)	0.0189 (3)	
H15C	1.1254	0.6816	0.6313	0.023*	
C16C	0.77946 (16)	0.85010 (14)	0.66691 (10)	0.0273 (4)	
H16E	0.7948	0.9047	0.6672	0.033*	
H16F	0.7469	0.8392	0.7068	0.033*	
C17C	0.69856 (16)	0.88136 (15)	0.60530 (10)	0.0282 (4)	
H17C	0.6871	0.8243	0.6035	0.034*	
C18C	0.7398 (2)	0.91101 (18)	0.54091 (11)	0.0405 (5)	
H18G	0.6890	0.9282	0.5038	0.061*	
H18H	0.8060	0.8560	0.5367	0.061*	
H18I	0.7504	0.9675	0.5417	0.061*	
C19C	0.59313 (18)	0.96733 (17)	0.61220 (12)	0.0396 (5)	
H19G	0.5427	0.9846	0.5748	0.059*	
H19H	0.6029	1.0241	0.6137	0.059*	
H19I	0.5672	0.9476	0.6525	0.059*	
C20C	1.10908 (14)	0.36409 (13)	0.47611 (8)	0.0187 (3)	
H20D	1.1839	0.3399	0.4739	0.028*	
H20E	1.0711	0.4320	0.4504	0.028*	
H20F	1.0865	0.3234	0.4586	0.028*	
C21C	1.26744 (14)	0.47668 (14)	0.71559 (9)	0.0240 (4)	
H21G	1.3315	0.4186	0.7127	0.036*	
H21H	1.2505	0.4696	0.7606	0.036*	
H21I	1.2774	0.5349	0.7023	0.036*	
Cl1D	0.33409 (4)	0.15331 (4)	0.67365 (3)	0.03099 (11)	
O1D	0.76092 (10)	0.52467 (10)	0.57158 (6)	0.0216 (2)	
N1D	0.60890 (11)	0.39985 (10)	0.58141 (7)	0.0167 (3)	
N2D	0.66647 (11)	0.44725 (11)	0.55537 (7)	0.0162 (3)	
C1D	0.52222 (15)	0.28151 (14)	0.64529 (8)	0.0222 (3)	
H1DA	0.5628	0.2965	0.6716	0.027*	
C2D	0.46518 (15)	0.23513 (14)	0.67528 (9)	0.0243 (4)	
H2DA	0.4670	0.2194	0.7213	0.029*	
C3D	0.40526 (13)	0.21239 (13)	0.63595 (9)	0.0212 (3)	
C4D	0.40132 (14)	0.23549 (13)	0.56744 (9)	0.0221 (3)	
H4DA	0.3605	0.2203	0.5415	0.026*	
C5D	0.45956 (14)	0.28198 (13)	0.53786 (8)	0.0198 (3)	
H5DA	0.4579	0.2970	0.4918	0.024*	
C6D	0.51998 (13)	0.30627 (12)	0.57588 (8)	0.0162 (3)	
C7D	0.58123 (13)	0.35672 (12)	0.54432 (8)	0.0157 (3)	
C8D	0.70674 (13)	0.48495 (12)	0.59466 (8)	0.0156 (3)	
C9D	0.68056 (13)	0.47965 (12)	0.66623 (8)	0.0155 (3)	
H9DA	0.6624	0.4253	0.6806	0.019*	
C10D	0.58518 (13)	0.57785 (12)	0.66919 (7)	0.0152 (3)	
C11D	0.49736 (14)	0.57821 (13)	0.69842 (8)	0.0191 (3)	
H11D	0.4962	0.5182	0.7154	0.023*	
C12D	0.41070 (14)	0.66837 (13)	0.70236 (9)	0.0221 (3)	
H12D	0.3532	0.6674	0.7229	0.027*	
C13D	0.40926 (14)	0.75970 (13)	0.67600 (8)	0.0207 (3)	
C14D	0.49810 (14)	0.75835 (13)	0.64741 (8)	0.0200 (3)	
H14D	0.4991	0.8183	0.6299	0.024*	
C15D	0.58506 (14)	0.66909 (12)	0.64464 (8)	0.0178 (3)	
H15D	0.6441	0.6700	0.6262	0.021*	
C16D	0.31442 (15)	0.85847 (14)	0.67582 (9)	0.0249 (4)	
H16G	0.3392	0.9051	0.6820	0.030*	
H16H	0.2764	0.8489	0.7132	0.030*	
C17D	0.23748 (15)	0.90435 (14)	0.61163 (10)	0.0257 (4)	
H17D	0.2782	0.9056	0.5737	0.031*	
C18D	0.15588 (19)	1.01160 (16)	0.61008 (12)	0.0377 (5)	
H18J	0.1115	1.0415	0.5683	0.056*	
H18K	0.1919	1.0493	0.6153	0.056*	
H18L	0.1130	1.0115	0.6457	0.056*	
C19D	0.18252 (18)	0.84273 (18)	0.60520 (13)	0.0386 (5)	
H19J	0.1406	0.8690	0.5627	0.058*	
H19K	0.1373	0.8456	0.6398	0.058*	
H19L	0.2348	0.7746	0.6091	0.058*	
C20D	0.60643 (14)	0.35489 (14)	0.47427 (8)	0.0198 (3)	
H20G	0.6786	0.3423	0.4714	0.030*	
H20H	0.5595	0.4182	0.4449	0.030*	
H20I	0.5973	0.3030	0.4617	0.030*	
C21D	0.77743 (14)	0.45816 (14)	0.71156 (9)	0.0224 (3)	
H21J	0.8364	0.3964	0.7083	0.034*	
H21K	0.7613	0.4532	0.7567	0.034*	
H21L	0.7954	0.5114	0.6981	0.034*	
H2NA	0.1426 (18)	1.0402 (17)	0.9845 (6)	0.026 (6)*	
H2NB	0.6339 (18)	1.0537 (17)	0.9789 (7)	0.029 (6)*	
H2NC	1.1837 (19)	0.4558 (17)	0.5201 (6)	0.030 (6)*	
H2ND	0.6834 (19)	0.4520 (18)	0.5157 (6)	0.033 (6)*	

### Atomic displacement parameters (Å^2^)

**Table:**

	*U*^11^	*U*^22^	*U*^33^	*U*^12^	*U*^13^	*U*^23^
Cl1A	0.0224 (2)	0.0330 (2)	0.0319 (2)	−0.01439 (19)	−0.00772 (17)	0.00239 (19)
O1A	0.0165 (6)	0.0284 (6)	0.0192 (6)	−0.0084 (5)	0.0015 (4)	−0.0086 (5)
N1A	0.0159 (6)	0.0157 (6)	0.0155 (6)	−0.0069 (5)	0.0012 (5)	−0.0037 (5)
N2A	0.0169 (6)	0.0178 (6)	0.0129 (6)	−0.0074 (5)	0.0014 (5)	−0.0057 (5)
C1A	0.0215 (8)	0.0206 (8)	0.0150 (7)	−0.0094 (6)	0.0018 (6)	−0.0034 (6)
C2A	0.0256 (9)	0.0204 (8)	0.0165 (7)	−0.0120 (7)	−0.0014 (6)	−0.0017 (6)
C3A	0.0195 (8)	0.0176 (7)	0.0223 (8)	−0.0101 (6)	−0.0040 (6)	0.0000 (6)
C4A	0.0189 (8)	0.0206 (8)	0.0217 (8)	−0.0106 (6)	0.0027 (6)	−0.0036 (6)
C5A	0.0193 (8)	0.0172 (7)	0.0150 (7)	−0.0083 (6)	0.0023 (6)	−0.0023 (6)
C6A	0.0178 (7)	0.0138 (6)	0.0141 (7)	−0.0076 (6)	0.0016 (5)	−0.0036 (5)
C7A	0.0184 (7)	0.0138 (6)	0.0137 (7)	−0.0076 (6)	0.0018 (5)	−0.0032 (5)
C8A	0.0182 (7)	0.0159 (7)	0.0151 (7)	−0.0086 (6)	0.0029 (5)	−0.0047 (5)
C9A	0.0190 (7)	0.0176 (7)	0.0117 (6)	−0.0085 (6)	0.0032 (5)	−0.0039 (5)
C10A	0.0191 (7)	0.0183 (7)	0.0108 (6)	−0.0086 (6)	0.0033 (5)	−0.0051 (5)
C11A	0.0220 (8)	0.0214 (8)	0.0165 (7)	−0.0097 (7)	−0.0021 (6)	−0.0026 (6)
C12A	0.0286 (9)	0.0262 (9)	0.0181 (8)	−0.0143 (7)	−0.0035 (7)	−0.0050 (7)
C13A	0.0330 (10)	0.0227 (8)	0.0152 (7)	−0.0140 (7)	0.0036 (6)	−0.0077 (6)
C14A	0.0271 (9)	0.0172 (7)	0.0185 (8)	−0.0069 (7)	0.0047 (6)	−0.0059 (6)
C15A	0.0177 (8)	0.0195 (7)	0.0162 (7)	−0.0069 (6)	0.0031 (6)	−0.0047 (6)
C16A	0.0422 (12)	0.0266 (9)	0.0231 (9)	−0.0187 (9)	0.0028 (8)	−0.0111 (7)
C17A	0.0372 (11)	0.0239 (9)	0.0254 (9)	−0.0172 (8)	−0.0003 (8)	−0.0049 (7)
C18A	0.0523 (15)	0.0344 (11)	0.0288 (10)	−0.0220 (11)	−0.0033 (10)	−0.0019 (9)
C19A	0.0495 (14)	0.0346 (11)	0.0420 (13)	−0.0270 (11)	0.0042 (10)	−0.0093 (10)
C2OA	0.0191 (8)	0.0220 (8)	0.0144 (7)	−0.0093 (6)	0.0027 (6)	−0.0069 (6)
C21A	0.0290 (9)	0.0260 (9)	0.0189 (8)	−0.0165 (7)	0.0088 (7)	−0.0070 (7)
Cl1B	0.0221 (2)	0.0328 (2)	0.0368 (3)	−0.01450 (19)	−0.01090 (18)	0.0050 (2)
O1B	0.0185 (6)	0.0274 (6)	0.0190 (6)	−0.0103 (5)	0.0027 (4)	−0.0091 (5)
N1B	0.0169 (6)	0.0181 (6)	0.0155 (6)	−0.0085 (5)	0.0019 (5)	−0.0050 (5)
N2B	0.0159 (6)	0.0205 (6)	0.0148 (6)	−0.0084 (5)	0.0021 (5)	−0.0067 (5)
C1B	0.0229 (9)	0.0263 (8)	0.0154 (7)	−0.0114 (7)	0.0017 (6)	−0.0041 (6)
C2B	0.0268 (9)	0.0274 (9)	0.0167 (8)	−0.0125 (7)	−0.0046 (6)	−0.0013 (7)
C3B	0.0202 (8)	0.0179 (7)	0.0255 (8)	−0.0107 (6)	−0.0048 (6)	−0.0003 (6)
C4B	0.0182 (8)	0.0243 (8)	0.0228 (8)	−0.0107 (7)	0.0015 (6)	−0.0006 (7)
C5B	0.0201 (8)	0.0235 (8)	0.0161 (7)	−0.0107 (7)	0.0014 (6)	−0.0019 (6)
C6B	0.0178 (7)	0.0165 (7)	0.0143 (7)	−0.0083 (6)	0.0007 (5)	−0.0041 (5)
C7B	0.0181 (7)	0.0178 (7)	0.0134 (7)	−0.0100 (6)	0.0021 (5)	−0.0041 (5)
C8B	0.0187 (7)	0.0159 (7)	0.0151 (7)	−0.0094 (6)	0.0036 (5)	−0.0052 (5)
C9B	0.0199 (8)	0.0164 (7)	0.0136 (7)	−0.0093 (6)	0.0032 (5)	−0.0043 (5)
C10B	0.0190 (7)	0.0168 (7)	0.0114 (6)	−0.0088 (6)	0.0032 (5)	−0.0044 (5)
C11B	0.0207 (8)	0.0163 (7)	0.0157 (7)	−0.0074 (6)	−0.0014 (6)	−0.0027 (6)
C12B	0.0258 (9)	0.0216 (8)	0.0193 (8)	−0.0125 (7)	−0.0034 (6)	−0.0053 (6)
C13B	0.0297 (9)	0.0174 (7)	0.0149 (7)	−0.0118 (7)	0.0023 (6)	−0.0060 (6)
C14B	0.0216 (8)	0.0177 (7)	0.0186 (7)	−0.0052 (6)	0.0028 (6)	−0.0052 (6)
C15B	0.0187 (8)	0.0199 (7)	0.0159 (7)	−0.0080 (6)	0.0032 (6)	−0.0049 (6)
C16B	0.0360 (10)	0.0186 (8)	0.0206 (8)	−0.0129 (7)	0.0020 (7)	−0.0081 (6)
C17B	0.0332 (10)	0.0222 (8)	0.0235 (8)	−0.0147 (8)	0.0032 (7)	−0.0076 (7)
C18B	0.0405 (13)	0.0385 (12)	0.0514 (14)	−0.0256 (10)	0.0184 (11)	−0.0202 (11)
C19B	0.0533 (14)	0.0237 (9)	0.0374 (12)	−0.0202 (10)	0.0067 (10)	−0.0079 (9)
C20B	0.0201 (8)	0.0280 (9)	0.0152 (7)	−0.0112 (7)	0.0027 (6)	−0.0083 (6)
C21B	0.0309 (10)	0.0268 (9)	0.0192 (8)	−0.0188 (8)	0.0100 (7)	−0.0071 (7)
Cl1C	0.0259 (2)	0.0361 (3)	0.0350 (3)	−0.0180 (2)	0.00643 (19)	0.0037 (2)
O1C	0.0241 (6)	0.0286 (6)	0.0199 (6)	−0.0161 (5)	0.0070 (5)	−0.0104 (5)
N1C	0.0168 (6)	0.0166 (6)	0.0154 (6)	−0.0076 (5)	0.0022 (5)	−0.0039 (5)
N2C	0.0193 (7)	0.0197 (6)	0.0139 (6)	−0.0101 (5)	0.0043 (5)	−0.0065 (5)
C1C	0.0196 (8)	0.0208 (8)	0.0145 (7)	−0.0083 (6)	0.0014 (6)	−0.0033 (6)
C2C	0.0207 (8)	0.0216 (8)	0.0162 (7)	−0.0072 (7)	0.0034 (6)	−0.0005 (6)
C3C	0.0169 (8)	0.0187 (7)	0.0234 (8)	−0.0077 (6)	0.0047 (6)	0.0001 (6)
C4C	0.0180 (8)	0.0217 (8)	0.0225 (8)	−0.0096 (7)	0.0000 (6)	−0.0020 (6)
C5C	0.0202 (8)	0.0180 (7)	0.0148 (7)	−0.0085 (6)	0.0006 (6)	−0.0015 (6)
C6C	0.0147 (7)	0.0143 (7)	0.0152 (7)	−0.0046 (6)	0.0023 (5)	−0.0029 (5)
C7C	0.0155 (7)	0.0148 (7)	0.0138 (7)	−0.0050 (6)	0.0014 (5)	−0.0032 (5)
C8C	0.0149 (7)	0.0166 (7)	0.0155 (7)	−0.0053 (6)	0.0010 (5)	−0.0049 (6)
C9C	0.0190 (8)	0.0174 (7)	0.0127 (6)	−0.0074 (6)	0.0010 (5)	−0.0050 (5)
C10C	0.0184 (7)	0.0165 (7)	0.0112 (6)	−0.0080 (6)	0.0012 (5)	−0.0034 (5)
C11C	0.0207 (8)	0.0197 (7)	0.0163 (7)	−0.0098 (6)	0.0039 (6)	−0.0034 (6)
C12C	0.0210 (8)	0.0250 (8)	0.0175 (7)	−0.0098 (7)	0.0066 (6)	−0.0047 (6)
C13C	0.0234 (8)	0.0211 (8)	0.0147 (7)	−0.0066 (7)	0.0045 (6)	−0.0065 (6)
C14C	0.0277 (9)	0.0175 (7)	0.0188 (8)	−0.0105 (7)	0.0039 (6)	−0.0047 (6)
C15C	0.0224 (8)	0.0201 (7)	0.0173 (7)	−0.0119 (7)	0.0039 (6)	−0.0060 (6)
C16C	0.0267 (10)	0.0237 (9)	0.0236 (9)	−0.0040 (7)	0.0066 (7)	−0.0086 (7)
C17C	0.0270 (10)	0.0227 (9)	0.0251 (9)	−0.0053 (7)	0.0040 (7)	−0.0018 (7)
C18C	0.0451 (14)	0.0347 (11)	0.0272 (10)	−0.0100 (10)	0.0062 (9)	0.0003 (9)
C19C	0.0311 (12)	0.0335 (11)	0.0411 (12)	−0.0056 (9)	0.0029 (9)	−0.0063 (9)
C20C	0.0221 (8)	0.0224 (8)	0.0155 (7)	−0.0129 (7)	0.0043 (6)	−0.0063 (6)
C21C	0.0206 (8)	0.0273 (9)	0.0195 (8)	−0.0062 (7)	−0.0026 (6)	−0.0084 (7)
Cl1D	0.0232 (2)	0.0314 (2)	0.0346 (2)	−0.01496 (19)	0.00650 (18)	0.00417 (19)
O1D	0.0254 (6)	0.0279 (6)	0.0191 (6)	−0.0172 (5)	0.0069 (5)	−0.0096 (5)
N1D	0.0170 (6)	0.0181 (6)	0.0153 (6)	−0.0080 (5)	0.0026 (5)	−0.0052 (5)
N2D	0.0184 (7)	0.0202 (6)	0.0134 (6)	−0.0107 (5)	0.0040 (5)	−0.0067 (5)
C1D	0.0257 (9)	0.0273 (9)	0.0146 (7)	−0.0135 (7)	0.0026 (6)	−0.0045 (6)
C2D	0.0258 (9)	0.0261 (9)	0.0180 (8)	−0.0112 (7)	0.0058 (7)	−0.0022 (7)
C3D	0.0148 (7)	0.0183 (7)	0.0246 (8)	−0.0053 (6)	0.0046 (6)	0.0004 (6)
C4D	0.0192 (8)	0.0238 (8)	0.0226 (8)	−0.0116 (7)	−0.0009 (6)	−0.0014 (7)
C5D	0.0206 (8)	0.0226 (8)	0.0165 (7)	−0.0116 (7)	0.0008 (6)	−0.0021 (6)
C6D	0.0165 (7)	0.0162 (7)	0.0151 (7)	−0.0066 (6)	0.0024 (5)	−0.0046 (6)
C7D	0.0153 (7)	0.0168 (7)	0.0140 (7)	−0.0066 (6)	0.0015 (5)	−0.0042 (5)
C8D	0.0156 (7)	0.0157 (7)	0.0149 (7)	−0.0058 (6)	0.0014 (5)	−0.0060 (5)
C9D	0.0164 (7)	0.0162 (7)	0.0130 (6)	−0.0065 (6)	0.0012 (5)	−0.0045 (5)
C10D	0.0172 (7)	0.0164 (7)	0.0109 (6)	−0.0066 (6)	0.0010 (5)	−0.0044 (5)
C11D	0.0205 (8)	0.0199 (7)	0.0159 (7)	−0.0096 (6)	0.0033 (6)	−0.0024 (6)
C12D	0.0204 (8)	0.0242 (8)	0.0190 (8)	−0.0082 (7)	0.0077 (6)	−0.0050 (6)
C13D	0.0218 (8)	0.0197 (8)	0.0159 (7)	−0.0048 (6)	0.0026 (6)	−0.0070 (6)
C14D	0.0261 (9)	0.0157 (7)	0.0174 (7)	−0.0088 (6)	0.0011 (6)	−0.0047 (6)
C15D	0.0201 (8)	0.0189 (7)	0.0159 (7)	−0.0104 (6)	0.0020 (6)	−0.0047 (6)
C16D	0.0274 (9)	0.0210 (8)	0.0220 (8)	−0.0059 (7)	0.0053 (7)	−0.0098 (7)
C17D	0.0229 (9)	0.0219 (8)	0.0251 (9)	−0.0039 (7)	0.0031 (7)	−0.0074 (7)
C18D	0.0357 (12)	0.0257 (10)	0.0373 (12)	−0.0025 (9)	−0.0004 (9)	−0.0080 (9)
C19D	0.0267 (11)	0.0384 (12)	0.0524 (14)	−0.0125 (9)	0.0043 (10)	−0.0209 (11)
C20D	0.0210 (8)	0.0281 (8)	0.0150 (7)	−0.0139 (7)	0.0041 (6)	−0.0090 (6)
C21D	0.0181 (8)	0.0246 (8)	0.0205 (8)	−0.0051 (7)	−0.0027 (6)	−0.0092 (7)

### Geometric parameters (Å, °)

**Table:**

Cl1A—C3A	1.7411 (18)	Cl1C—C3C	1.7396 (17)
O1A—C8A	1.230 (2)	O1C—C8C	1.234 (2)
N1A—C7A	1.288 (2)	N1C—C7C	1.290 (2)
N1A—N2A	1.3720 (19)	N1C—N2C	1.3766 (19)
N2A—C8A	1.358 (2)	N2C—C8C	1.361 (2)
N2A—H2NA	0.846 (9)	N2C—H2NC	0.845 (9)
C1A—C2A	1.383 (2)	C1C—C2C	1.389 (2)
C1A—C6A	1.399 (2)	C1C—C6C	1.400 (2)
C1A—H1AA	0.9300	C1C—H1CA	0.9300
C2A—C3A	1.391 (3)	C2C—C3C	1.386 (3)
C2A—H2AA	0.9300	C2C—H2CA	0.9300
C3A—C4A	1.382 (2)	C3C—C4C	1.384 (2)
C4A—C5A	1.390 (2)	C4C—C5C	1.393 (2)
C4A—H4AA	0.9300	C4C—H4CA	0.9300
C5A—C6A	1.399 (2)	C5C—C6C	1.398 (2)
C5A—H5AA	0.9300	C5C—H5CA	0.9300
C6A—C7A	1.487 (2)	C6C—C7C	1.485 (2)
C7A—C2OA	1.506 (2)	C7C—C20C	1.502 (2)
C8A—C9A	1.525 (2)	C8C—C9C	1.520 (2)
C9A—C10A	1.526 (2)	C9C—C10C	1.523 (2)
C9A—C21A	1.531 (2)	C9C—C21C	1.534 (2)
C9A—H9AA	0.9800	C9C—H9CA	0.9800
C10A—C11A	1.396 (2)	C10C—C15C	1.396 (2)
C10A—C15A	1.398 (2)	C10C—C11C	1.399 (2)
C11A—C12A	1.397 (2)	C11C—C12C	1.388 (2)
C11A—H11A	0.9300	C11C—H11C	0.9300
C12A—C13A	1.390 (3)	C12C—C13C	1.395 (2)
C12A—H12A	0.9300	C12C—H12C	0.9300
C13A—C14A	1.403 (3)	C13C—C14C	1.402 (3)
C13A—C16A	1.513 (3)	C13C—C16C	1.508 (2)
C14A—C15A	1.390 (2)	C14C—C15C	1.392 (2)
C14A—H14A	0.9300	C14C—H14C	0.9300
C15A—H15A	0.9300	C15C—H15C	0.9300
C16A—C17A	1.543 (3)	C16C—C17C	1.554 (3)
C16A—H16A	0.9700	C16C—H16E	0.9700
C16A—H16B	0.9700	C16C—H16F	0.9700
C17A—C18A	1.526 (3)	C17C—C19C	1.519 (3)
C17A—C19A	1.530 (3)	C17C—C18C	1.525 (3)
C17A—H17A	0.9800	C17C—H17C	0.9800
C18A—H18A	0.9600	C18C—H18G	0.9600
C18A—H18B	0.9600	C18C—H18H	0.9600
C18A—H18C	0.9600	C18C—H18I	0.9600
C19A—H19A	0.9600	C19C—H19G	0.9600
C19A—H19B	0.9600	C19C—H19H	0.9600
C19A—H19C	0.9600	C19C—H19I	0.9600
C2OA—H2OA	0.9600	C20C—H20D	0.9600
C2OA—H2OB	0.9600	C20C—H20E	0.9600
C2OA—H2OC	0.9600	C20C—H20F	0.9600
C21A—H21A	0.9600	C21C—H21G	0.9600
C21A—H21B	0.9600	C21C—H21H	0.9600
C21A—H21C	0.9600	C21C—H21I	0.9600
Cl1B—C3B	1.7383 (18)	Cl1D—C3D	1.7403 (18)
O1B—C8B	1.230 (2)	O1D—C8D	1.232 (2)
N1B—C7B	1.290 (2)	N1D—C7D	1.290 (2)
N1B—N2B	1.379 (2)	N1D—N2D	1.3752 (19)
N2B—C8B	1.361 (2)	N2D—C8D	1.361 (2)
N2B—H2NB	0.853 (9)	N2D—H2ND	0.855 (9)
C1B—C2B	1.383 (3)	C1D—C2D	1.383 (2)
C1B—C6B	1.406 (2)	C1D—C6D	1.403 (2)
C1B—H1BA	0.9300	C1D—H1DA	0.9300
C2B—C3B	1.384 (3)	C2D—C3D	1.386 (3)
C2B—H2BA	0.9300	C2D—H2DA	0.9300
C3B—C4B	1.385 (3)	C3D—C4D	1.382 (2)
C4B—C5B	1.391 (2)	C4D—C5D	1.395 (2)
C4B—H4BA	0.9300	C4D—H4DA	0.9300
C5B—C6B	1.394 (2)	C5D—C6D	1.391 (2)
C5B—H5BA	0.9300	C5D—H5DA	0.9300
C6B—C7B	1.481 (2)	C6D—C7D	1.487 (2)
C7B—C20B	1.503 (2)	C7D—C20D	1.501 (2)
C8B—C9B	1.525 (2)	C8D—C9D	1.525 (2)
C9B—C10B	1.521 (2)	C9D—C10D	1.523 (2)
C9B—C21B	1.538 (2)	C9D—C21D	1.531 (2)
C9B—H9BA	0.9800	C9D—H9DA	0.9800
C10B—C11B	1.392 (2)	C10D—C11D	1.391 (2)
C10B—C15B	1.396 (2)	C10D—C15D	1.397 (2)
C11B—C12B	1.396 (2)	C11D—C12D	1.400 (2)
C11B—H11B	0.9300	C11D—H11D	0.9300
C12B—C13B	1.395 (2)	C12D—C13D	1.395 (3)
C12B—H12B	0.9300	C12D—H12D	0.9300
C13B—C14B	1.395 (3)	C13D—C14D	1.393 (3)
C13B—C16B	1.508 (2)	C13D—C16D	1.510 (2)
C14B—C15B	1.391 (2)	C14D—C15D	1.387 (2)
C14B—H14B	0.9300	C14D—H14D	0.9300
C15B—H15B	0.9300	C15D—H15D	0.9300
C16B—C17B	1.530 (3)	C16D—C17D	1.534 (3)
C16B—H16C	0.9700	C16D—H16G	0.9700
C16B—H16D	0.9700	C16D—H16H	0.9700
C17B—C18B	1.517 (3)	C17D—C19D	1.514 (3)
C17B—C19B	1.528 (3)	C17D—C18D	1.527 (3)
C17B—H17B	0.9800	C17D—H17D	0.9800
C18B—H18D	0.9600	C18D—H18J	0.9600
C18B—H18E	0.9600	C18D—H18K	0.9600
C18B—H18F	0.9600	C18D—H18L	0.9600
C19B—H19D	0.9600	C19D—H19J	0.9600
C19B—H19E	0.9600	C19D—H19K	0.9600
C19B—H19F	0.9600	C19D—H19L	0.9600
C20B—H20A	0.9600	C20D—H20G	0.9600
C20B—H20B	0.9600	C20D—H20H	0.9600
C20B—H20C	0.9600	C20D—H20I	0.9600
C21B—H21D	0.9600	C21D—H21J	0.9600
C21B—H21E	0.9600	C21D—H21K	0.9600
C21B—H21F	0.9600	C21D—H21L	0.9600
			
C7A—N1A—N2A	117.79 (14)	C7C—N1C—N2C	117.50 (13)
C8A—N2A—N1A	120.07 (13)	C8C—N2C—N1C	120.35 (13)
C8A—N2A—H2NA	116.6 (16)	C8C—N2C—H2NC	118.2 (16)
N1A—N2A—H2NA	123.3 (16)	N1C—N2C—H2NC	121.5 (16)
C2A—C1A—C6A	121.00 (16)	C2C—C1C—C6C	120.73 (16)
C2A—C1A—H1AA	119.5	C2C—C1C—H1CA	119.6
C6A—C1A—H1AA	119.5	C6C—C1C—H1CA	119.6
C1A—C2A—C3A	119.23 (16)	C3C—C2C—C1C	119.57 (16)
C1A—C2A—H2AA	120.4	C3C—C2C—H2CA	120.2
C3A—C2A—H2AA	120.4	C1C—C2C—H2CA	120.2
C4A—C3A—C2A	121.09 (16)	C4C—C3C—C2C	120.95 (16)
C4A—C3A—Cl1A	119.70 (14)	C4C—C3C—Cl1C	119.61 (14)
C2A—C3A—Cl1A	119.21 (13)	C2C—C3C—Cl1C	119.43 (13)
C3A—C4A—C5A	119.27 (16)	C3C—C4C—C5C	119.29 (17)
C3A—C4A—H4AA	120.4	C3C—C4C—H4CA	120.4
C5A—C4A—H4AA	120.4	C5C—C4C—H4CA	120.4
C4A—C5A—C6A	120.87 (15)	C4C—C5C—C6C	120.86 (15)
C4A—C5A—H5AA	119.6	C4C—C5C—H5CA	119.6
C6A—C5A—H5AA	119.6	C6C—C5C—H5CA	119.6
C5A—C6A—C1A	118.54 (15)	C5C—C6C—C1C	118.59 (15)
C5A—C6A—C7A	121.23 (14)	C5C—C6C—C7C	121.12 (14)
C1A—C6A—C7A	120.22 (14)	C1C—C6C—C7C	120.28 (15)
N1A—C7A—C6A	115.10 (14)	N1C—C7C—C6C	115.22 (14)
N1A—C7A—C2OA	124.00 (15)	N1C—C7C—C20C	124.27 (15)
C6A—C7A—C2OA	120.90 (14)	C6C—C7C—C20C	120.50 (14)
O1A—C8A—N2A	119.99 (15)	O1C—C8C—N2C	119.73 (15)
O1A—C8A—C9A	121.55 (14)	O1C—C8C—C9C	121.88 (15)
N2A—C8A—C9A	118.44 (14)	N2C—C8C—C9C	118.36 (14)
C8A—C9A—C10A	108.95 (13)	C8C—C9C—C10C	108.80 (13)
C8A—C9A—C21A	109.38 (14)	C8C—C9C—C21C	109.77 (14)
C10A—C9A—C21A	112.13 (13)	C10C—C9C—C21C	111.96 (13)
C8A—C9A—H9AA	108.8	C8C—C9C—H9CA	108.8
C10A—C9A—H9AA	108.8	C10C—C9C—H9CA	108.8
C21A—C9A—H9AA	108.8	C21C—C9C—H9CA	108.8
C11A—C10A—C15A	118.27 (15)	C15C—C10C—C11C	118.21 (15)
C11A—C10A—C9A	120.98 (15)	C15C—C10C—C9C	120.54 (15)
C15A—C10A—C9A	120.75 (15)	C11C—C10C—C9C	121.25 (14)
C10A—C11A—C12A	120.76 (16)	C12C—C11C—C10C	120.78 (16)
C10A—C11A—H11A	119.6	C12C—C11C—H11C	119.6
C12A—C11A—H11A	119.6	C10C—C11C—H11C	119.6
C13A—C12A—C11A	121.30 (17)	C11C—C12C—C13C	121.50 (16)
C13A—C12A—H12A	119.4	C11C—C12C—H12C	119.2
C11A—C12A—H12A	119.4	C13C—C12C—H12C	119.2
C12A—C13A—C14A	117.62 (16)	C12C—C13C—C14C	117.50 (16)
C12A—C13A—C16A	120.80 (18)	C12C—C13C—C16C	120.90 (17)
C14A—C13A—C16A	121.57 (17)	C14C—C13C—C16C	121.58 (17)
C15A—C14A—C13A	121.42 (17)	C15C—C14C—C13C	121.25 (16)
C15A—C14A—H14A	119.3	C15C—C14C—H14C	119.4
C13A—C14A—H14A	119.3	C13C—C14C—H14C	119.4
C14A—C15A—C10A	120.61 (16)	C14C—C15C—C10C	120.75 (16)
C14A—C15A—H15A	119.7	C14C—C15C—H15C	119.6
C10A—C15A—H15A	119.7	C10C—C15C—H15C	119.6
C13A—C16A—C17A	114.33 (15)	C13C—C16C—C17C	114.18 (15)
C13A—C16A—H16A	108.7	C13C—C16C—H16E	108.7
C17A—C16A—H16A	108.7	C17C—C16C—H16E	108.7
C13A—C16A—H16B	108.7	C13C—C16C—H16F	108.7
C17A—C16A—H16B	108.7	C17C—C16C—H16F	108.7
H16A—C16A—H16B	107.6	H16E—C16C—H16F	107.6
C18A—C17A—C19A	109.84 (18)	C19C—C17C—C18C	110.07 (18)
C18A—C17A—C16A	112.05 (18)	C19C—C17C—C16C	109.86 (17)
C19A—C17A—C16A	109.77 (17)	C18C—C17C—C16C	111.50 (18)
C18A—C17A—H17A	108.4	C19C—C17C—H17C	108.4
C19A—C17A—H17A	108.4	C18C—C17C—H17C	108.4
C16A—C17A—H17A	108.4	C16C—C17C—H17C	108.4
C17A—C18A—H18A	109.5	C17C—C18C—H18G	109.5
C17A—C18A—H18B	109.5	C17C—C18C—H18H	109.5
H18A—C18A—H18B	109.5	H18G—C18C—H18H	109.5
C17A—C18A—H18C	109.5	C17C—C18C—H18I	109.5
H18A—C18A—H18C	109.5	H18G—C18C—H18I	109.5
H18B—C18A—H18C	109.5	H18H—C18C—H18I	109.5
C17A—C19A—H19A	109.5	C17C—C19C—H19G	109.5
C17A—C19A—H19B	109.5	C17C—C19C—H19H	109.5
H19A—C19A—H19B	109.5	H19G—C19C—H19H	109.5
C17A—C19A—H19C	109.5	C17C—C19C—H19I	109.5
H19A—C19A—H19C	109.5	H19G—C19C—H19I	109.5
H19B—C19A—H19C	109.5	H19H—C19C—H19I	109.5
C7A—C2OA—H2OA	109.5	C7C—C20C—H20D	109.5
C7A—C2OA—H2OB	109.5	C7C—C20C—H20E	109.5
H2OA—C2OA—H2OB	109.5	H20D—C20C—H20E	109.5
C7A—C2OA—H2OC	109.5	C7C—C20C—H20F	109.5
H2OA—C2OA—H2OC	109.5	H20D—C20C—H20F	109.5
H2OB—C2OA—H2OC	109.5	H20E—C20C—H20F	109.5
C9A—C21A—H21A	109.5	C9C—C21C—H21G	109.5
C9A—C21A—H21B	109.5	C9C—C21C—H21H	109.5
H21A—C21A—H21B	109.5	H21G—C21C—H21H	109.5
C9A—C21A—H21C	109.5	C9C—C21C—H21I	109.5
H21A—C21A—H21C	109.5	H21G—C21C—H21I	109.5
H21B—C21A—H21C	109.5	H21H—C21C—H21I	109.5
C7B—N1B—N2B	117.65 (14)	C7D—N1D—N2D	117.98 (13)
C8B—N2B—N1B	120.33 (14)	C8D—N2D—N1D	120.11 (13)
C8B—N2B—H2NB	115.2 (16)	C8D—N2D—H2ND	115.2 (17)
N1B—N2B—H2NB	124.0 (16)	N1D—N2D—H2ND	124.5 (17)
C2B—C1B—C6B	121.02 (16)	C2D—C1D—C6D	121.11 (17)
C2B—C1B—H1BA	119.5	C2D—C1D—H1DA	119.4
C6B—C1B—H1BA	119.5	C6D—C1D—H1DA	119.4
C1B—C2B—C3B	119.42 (16)	C1D—C2D—C3D	119.26 (16)
C1B—C2B—H2BA	120.3	C1D—C2D—H2DA	120.4
C3B—C2B—H2BA	120.3	C3D—C2D—H2DA	120.4
C2B—C3B—C4B	121.10 (16)	C4D—C3D—C2D	121.22 (16)
C2B—C3B—Cl1B	119.45 (14)	C4D—C3D—Cl1D	119.47 (15)
C4B—C3B—Cl1B	119.45 (14)	C2D—C3D—Cl1D	119.31 (14)
C3B—C4B—C5B	119.09 (17)	C3D—C4D—C5D	118.91 (17)
C3B—C4B—H4BA	120.5	C3D—C4D—H4DA	120.5
C5B—C4B—H4BA	120.5	C5D—C4D—H4DA	120.5
C4B—C5B—C6B	121.24 (16)	C6D—C5D—C4D	121.28 (16)
C4B—C5B—H5BA	119.4	C6D—C5D—H5DA	119.4
C6B—C5B—H5BA	119.4	C4D—C5D—H5DA	119.4
C5B—C6B—C1B	118.13 (16)	C5D—C6D—C1D	118.21 (15)
C5B—C6B—C7B	121.43 (14)	C5D—C6D—C7D	121.30 (14)
C1B—C6B—C7B	120.44 (15)	C1D—C6D—C7D	120.49 (15)
N1B—C7B—C6B	115.27 (14)	N1D—C7D—C6D	115.06 (14)
N1B—C7B—C20B	124.72 (15)	N1D—C7D—C20D	124.86 (15)
C6B—C7B—C20B	120.01 (14)	C6D—C7D—C20D	120.08 (14)
O1B—C8B—N2B	119.82 (15)	O1D—C8D—N2D	119.81 (14)
O1B—C8B—C9B	121.44 (14)	O1D—C8D—C9D	121.39 (14)
N2B—C8B—C9B	118.70 (14)	N2D—C8D—C9D	118.77 (14)
C10B—C9B—C8B	108.88 (13)	C10D—C9D—C8D	109.06 (13)
C10B—C9B—C21B	111.48 (13)	C10D—C9D—C21D	111.33 (13)
C8B—C9B—C21B	109.86 (14)	C8D—C9D—C21D	109.66 (14)
C10B—C9B—H9BA	108.9	C10D—C9D—H9DA	108.9
C8B—C9B—H9BA	108.9	C8D—C9D—H9DA	108.9
C21B—C9B—H9BA	108.9	C21D—C9D—H9DA	108.9
C11B—C10B—C15B	118.49 (15)	C11D—C10D—C15D	118.49 (15)
C11B—C10B—C9B	120.60 (15)	C11D—C10D—C9D	120.46 (14)
C15B—C10B—C9B	120.88 (15)	C15D—C10D—C9D	121.02 (14)
C10B—C11B—C12B	120.59 (16)	C10D—C11D—C12D	120.40 (16)
C10B—C11B—H11B	119.7	C10D—C11D—H11D	119.8
C12B—C11B—H11B	119.7	C12D—C11D—H11D	119.8
C13B—C12B—C11B	121.11 (16)	C13D—C12D—C11D	121.07 (16)
C13B—C12B—H12B	119.4	C13D—C12D—H12D	119.5
C11B—C12B—H12B	119.4	C11D—C12D—H12D	119.5
C12B—C13B—C14B	117.95 (15)	C14D—C13D—C12D	118.00 (16)
C12B—C13B—C16B	122.14 (17)	C14D—C13D—C16D	119.62 (16)
C14B—C13B—C16B	119.90 (16)	C12D—C13D—C16D	122.35 (17)
C15B—C14B—C13B	121.14 (16)	C15D—C14D—C13D	121.12 (16)
C15B—C14B—H14B	119.4	C15D—C14D—H14D	119.4
C13B—C14B—H14B	119.4	C13D—C14D—H14D	119.4
C14B—C15B—C10B	120.70 (16)	C14D—C15D—C10D	120.87 (16)
C14B—C15B—H15B	119.6	C14D—C15D—H15D	119.6
C10B—C15B—H15B	119.6	C10D—C15D—H15D	119.6
C13B—C16B—C17B	113.53 (14)	C13D—C16D—C17D	113.48 (15)
C13B—C16B—H16C	108.9	C13D—C16D—H16G	108.9
C17B—C16B—H16C	108.9	C17D—C16D—H16G	108.9
C13B—C16B—H16D	108.9	C13D—C16D—H16H	108.9
C17B—C16B—H16D	108.9	C17D—C16D—H16H	108.9
H16C—C16B—H16D	107.7	H16G—C16D—H16H	107.7
C18B—C17B—C19B	110.00 (18)	C19D—C17D—C18D	110.44 (18)
C18B—C17B—C16B	111.07 (17)	C19D—C17D—C16D	111.14 (17)
C19B—C17B—C16B	110.41 (16)	C18D—C17D—C16D	109.97 (16)
C18B—C17B—H17B	108.4	C19D—C17D—H17D	108.4
C19B—C17B—H17B	108.4	C18D—C17D—H17D	108.4
C16B—C17B—H17B	108.4	C16D—C17D—H17D	108.4
C17B—C18B—H18D	109.5	C17D—C18D—H18J	109.5
C17B—C18B—H18E	109.5	C17D—C18D—H18K	109.5
H18D—C18B—H18E	109.5	H18J—C18D—H18K	109.5
C17B—C18B—H18F	109.5	C17D—C18D—H18L	109.5
H18D—C18B—H18F	109.5	H18J—C18D—H18L	109.5
H18E—C18B—H18F	109.5	H18K—C18D—H18L	109.5
C17B—C19B—H19D	109.5	C17D—C19D—H19J	109.5
C17B—C19B—H19E	109.5	C17D—C19D—H19K	109.5
H19D—C19B—H19E	109.5	H19J—C19D—H19K	109.5
C17B—C19B—H19F	109.5	C17D—C19D—H19L	109.5
H19D—C19B—H19F	109.5	H19J—C19D—H19L	109.5
H19E—C19B—H19F	109.5	H19K—C19D—H19L	109.5
C7B—C20B—H20A	109.5	C7D—C20D—H20G	109.5
C7B—C20B—H20B	109.5	C7D—C20D—H20H	109.5
H20A—C20B—H20B	109.5	H20G—C20D—H20H	109.5
C7B—C20B—H20C	109.5	C7D—C20D—H20I	109.5
H20A—C20B—H20C	109.5	H20G—C20D—H20I	109.5
H20B—C20B—H20C	109.5	H20H—C20D—H20I	109.5
C9B—C21B—H21D	109.5	C9D—C21D—H21J	109.5
C9B—C21B—H21E	109.5	C9D—C21D—H21K	109.5
H21D—C21B—H21E	109.5	H21J—C21D—H21K	109.5
C9B—C21B—H21F	109.5	C9D—C21D—H21L	109.5
H21D—C21B—H21F	109.5	H21J—C21D—H21L	109.5
H21E—C21B—H21F	109.5	H21K—C21D—H21L	109.5
			
C7A—N1A—N2A—C8A	−177.18 (15)	C7C—N1C—N2C—C8C	−178.36 (15)
C6A—C1A—C2A—C3A	−0.1 (3)	C6C—C1C—C2C—C3C	−0.1 (3)
C1A—C2A—C3A—C4A	0.6 (3)	C1C—C2C—C3C—C4C	0.6 (3)
C1A—C2A—C3A—Cl1A	−179.72 (13)	C1C—C2C—C3C—Cl1C	−179.01 (13)
C2A—C3A—C4A—C5A	−0.3 (3)	C2C—C3C—C4C—C5C	−0.3 (3)
Cl1A—C3A—C4A—C5A	179.97 (13)	Cl1C—C3C—C4C—C5C	179.27 (13)
C3A—C4A—C5A—C6A	−0.4 (3)	C3C—C4C—C5C—C6C	−0.5 (3)
C4A—C5A—C6A—C1A	0.9 (2)	C4C—C5C—C6C—C1C	0.9 (2)
C4A—C5A—C6A—C7A	−177.91 (15)	C4C—C5C—C6C—C7C	−177.80 (15)
C2A—C1A—C6A—C5A	−0.7 (2)	C2C—C1C—C6C—C5C	−0.7 (2)
C2A—C1A—C6A—C7A	178.17 (15)	C2C—C1C—C6C—C7C	178.07 (15)
N2A—N1A—C7A—C6A	−179.62 (13)	N2C—N1C—C7C—C6C	−179.68 (13)
N2A—N1A—C7A—C2OA	−0.5 (2)	N2C—N1C—C7C—C20C	−0.1 (2)
C5A—C6A—C7A—N1A	158.74 (15)	C5C—C6C—C7C—N1C	157.33 (15)
C1A—C6A—C7A—N1A	−20.1 (2)	C1C—C6C—C7C—N1C	−21.4 (2)
C5A—C6A—C7A—C2OA	−20.4 (2)	C5C—C6C—C7C—C20C	−22.3 (2)
C1A—C6A—C7A—C2OA	160.83 (15)	C1C—C6C—C7C—C20C	159.02 (15)
N1A—N2A—C8A—O1A	178.98 (15)	N1C—N2C—C8C—O1C	177.82 (15)
N1A—N2A—C8A—C9A	−2.6 (2)	N1C—N2C—C8C—C9C	−4.0 (2)
O1A—C8A—C9A—C10A	86.39 (19)	O1C—C8C—C9C—C10C	86.41 (19)
N2A—C8A—C9A—C10A	−92.00 (17)	N2C—C8C—C9C—C10C	−91.70 (17)
O1A—C8A—C9A—C21A	−36.5 (2)	O1C—C8C—C9C—C21C	−36.4 (2)
N2A—C8A—C9A—C21A	145.12 (15)	N2C—C8C—C9C—C21C	145.47 (15)
C8A—C9A—C10A—C11A	122.54 (16)	C8C—C9C—C10C—C15C	−58.63 (19)
C21A—C9A—C10A—C11A	−116.24 (17)	C21C—C9C—C10C—C15C	62.9 (2)
C8A—C9A—C10A—C15A	−57.43 (19)	C8C—C9C—C10C—C11C	121.97 (16)
C21A—C9A—C10A—C15A	63.79 (19)	C21C—C9C—C10C—C11C	−116.53 (17)
C15A—C10A—C11A—C12A	0.4 (2)	C15C—C10C—C11C—C12C	−0.1 (2)
C9A—C10A—C11A—C12A	−179.53 (15)	C9C—C10C—C11C—C12C	179.35 (15)
C10A—C11A—C12A—C13A	0.9 (3)	C10C—C11C—C12C—C13C	0.9 (3)
C11A—C12A—C13A—C14A	−1.8 (3)	C11C—C12C—C13C—C14C	−1.2 (3)
C11A—C12A—C13A—C16A	176.77 (17)	C11C—C12C—C13C—C16C	177.22 (17)
C12A—C13A—C14A—C15A	1.4 (3)	C12C—C13C—C14C—C15C	0.7 (3)
C16A—C13A—C14A—C15A	−177.12 (16)	C16C—C13C—C14C—C15C	−177.70 (17)
C13A—C14A—C15A—C10A	−0.2 (3)	C13C—C14C—C15C—C10C	0.1 (3)
C11A—C10A—C15A—C14A	−0.8 (2)	C11C—C10C—C15C—C14C	−0.4 (2)
C9A—C10A—C15A—C14A	179.17 (15)	C9C—C10C—C15C—C14C	−179.84 (15)
C12A—C13A—C16A—C17A	−83.3 (2)	C12C—C13C—C16C—C17C	−81.4 (2)
C14A—C13A—C16A—C17A	95.2 (2)	C14C—C13C—C16C—C17C	96.9 (2)
C13A—C16A—C17A—C18A	−64.7 (2)	C13C—C16C—C17C—C19C	172.86 (18)
C13A—C16A—C17A—C19A	173.01 (18)	C13C—C16C—C17C—C18C	−64.8 (2)
C7B—N1B—N2B—C8B	−174.93 (15)	C7D—N1D—N2D—C8D	−173.29 (15)
C6B—C1B—C2B—C3B	0.3 (3)	C6D—C1D—C2D—C3D	0.3 (3)
C1B—C2B—C3B—C4B	0.2 (3)	C1D—C2D—C3D—C4D	−0.2 (3)
C1B—C2B—C3B—Cl1B	−179.83 (14)	C1D—C2D—C3D—Cl1D	179.60 (14)
C2B—C3B—C4B—C5B	−0.4 (3)	C2D—C3D—C4D—C5D	0.4 (3)
Cl1B—C3B—C4B—C5B	179.64 (14)	Cl1D—C3D—C4D—C5D	−179.39 (14)
C3B—C4B—C5B—C6B	0.0 (3)	C3D—C4D—C5D—C6D	−0.8 (3)
C4B—C5B—C6B—C1B	0.5 (3)	C4D—C5D—C6D—C1D	0.9 (3)
C4B—C5B—C6B—C7B	−179.38 (16)	C4D—C5D—C6D—C7D	−179.39 (16)
C2B—C1B—C6B—C5B	−0.7 (3)	C2D—C1D—C6D—C5D	−0.6 (3)
C2B—C1B—C6B—C7B	179.19 (16)	C2D—C1D—C6D—C7D	179.61 (16)
N2B—N1B—C7B—C6B	179.48 (13)	N2D—N1D—C7D—C6D	179.51 (14)
N2B—N1B—C7B—C20B	−0.1 (2)	N2D—N1D—C7D—C20D	−0.5 (2)
C5B—C6B—C7B—N1B	159.53 (16)	C5D—C6D—C7D—N1D	161.40 (16)
C1B—C6B—C7B—N1B	−20.3 (2)	C1D—C6D—C7D—N1D	−18.9 (2)
C5B—C6B—C7B—C20B	−20.8 (2)	C5D—C6D—C7D—C20D	−18.6 (2)
C1B—C6B—C7B—C20B	159.34 (16)	C1D—C6D—C7D—C20D	161.14 (16)
N1B—N2B—C8B—O1B	176.82 (15)	N1D—N2D—C8D—O1D	177.83 (15)
N1B—N2B—C8B—C9B	−5.5 (2)	N1D—N2D—C8D—C9D	−4.0 (2)
O1B—C8B—C9B—C10B	83.14 (19)	O1D—C8D—C9D—C10D	83.06 (19)
N2B—C8B—C9B—C10B	−94.54 (17)	N2D—C8D—C9D—C10D	−95.07 (17)
O1B—C8B—C9B—C21B	−39.2 (2)	O1D—C8D—C9D—C21D	−39.1 (2)
N2B—C8B—C9B—C21B	143.12 (15)	N2D—C8D—C9D—C21D	142.78 (15)
C8B—C9B—C10B—C11B	125.80 (16)	C8D—C9D—C10D—C11D	127.66 (16)
C21B—C9B—C10B—C11B	−112.85 (17)	C21D—C9D—C10D—C11D	−111.21 (17)
C8B—C9B—C10B—C15B	−56.36 (19)	C8D—C9D—C10D—C15D	−54.34 (19)
C21B—C9B—C10B—C15B	65.00 (19)	C21D—C9D—C10D—C15D	66.79 (19)
C15B—C10B—C11B—C12B	0.0 (2)	C15D—C10D—C11D—C12D	0.6 (2)
C9B—C10B—C11B—C12B	177.93 (15)	C9D—C10D—C11D—C12D	178.65 (15)
C10B—C11B—C12B—C13B	1.4 (3)	C10D—C11D—C12D—C13D	1.4 (3)
C11B—C12B—C13B—C14B	−1.5 (3)	C11D—C12D—C13D—C14D	−1.9 (3)
C11B—C12B—C13B—C16B	177.09 (16)	C11D—C12D—C13D—C16D	176.29 (17)
C12B—C13B—C14B—C15B	0.2 (3)	C12D—C13D—C14D—C15D	0.5 (3)
C16B—C13B—C14B—C15B	−178.42 (16)	C16D—C13D—C14D—C15D	−177.71 (16)
C13B—C14B—C15B—C10B	1.2 (3)	C13D—C14D—C15D—C10D	1.4 (3)
C11B—C10B—C15B—C14B	−1.3 (2)	C11D—C10D—C15D—C14D	−2.0 (2)
C9B—C10B—C15B—C14B	−179.21 (15)	C9D—C10D—C15D—C14D	179.99 (15)
C12B—C13B—C16B—C17B	−94.0 (2)	C14D—C13D—C16D—C17D	85.2 (2)
C14B—C13B—C16B—C17B	84.6 (2)	C12D—C13D—C16D—C17D	−93.0 (2)
C13B—C16B—C17B—C18B	68.2 (2)	C13D—C16D—C17D—C19D	67.9 (2)
C13B—C16B—C17B—C19B	−169.45 (18)	C13D—C16D—C17D—C18D	−169.53 (18)

### Hydrogen-bond geometry (Å, °)

**Table:**

*D*—H···*A*	*D*—H	H···*A*	*D*···*A*	*D*—H···*A*
N2A—H2NA···O1B^i^	0.85 (1)	2.09 (2)	2.9255 (19)	169 (3)
N2B—H2NB···O1A^i^	0.85 (2)	2.07 (2)	2.916 (2)	173 (2)
N2C—H2NC···O1D^ii^	0.85 (2)	2.08 (2)	2.9085 (19)	169 (3)
N2D—H2ND···O1Ci	0.86 (2)	2.09 (2)	2.934 (2)	172 (3)
C20B—H20A···O1A^i^	0.96	2.47	3.125 (3)	126
C20C—H20D···O1D^ii^	0.96	2.57	3.099 (3)	115
C20D—H20G···O1C^ii^	0.96	2.50	3.175 (3)	128
C2OA—H2OC···Cg1^iii^	0.96	2.75	3.4628 (19)	131
C16C—H16F···Cg2	0.96	2.78	3.744 (2)	171
C20B—H20C···Cg2^i^	0.96	2.94	3.5787 (19)	125
C16A—H16B···Cg3^iv^	0.96	2.90	3.860 (2)	170
C20C—H20F···Cg3^ii^	0.96	2.77	3.4943 (19)	132
C16B—H16D···Cg4	0.96	2.86	3.818 (2)	171
C20D—H20I···Cg4^v^	0.96	2.96	3.5557 (19)	122

Symmetry codes: (i) −*x*+1, −*y*+2, −*z*+2; (ii) −*x*+2, −*y*+1, −*z*+1; i; (iii) −*x*, −*y*+2, −*z*+2;  (iv) *x*−1, *y*, *z*; (v) −*x*+1, −*y*+1, −*z*+1.
